# Topical minoxidil and dietary supplement for the treatment of chemotherapy-induced alopecia in childhood: a retrospective cohort study

**DOI:** 10.1038/s41598-024-53054-8

**Published:** 2024-02-22

**Authors:** Ji Won Lee, Jeewoo Kang, Jung Yoon Choi, Kyung Taek Hong, Hyoung Jin Kang, Ohsang Kwon

**Affiliations:** 1 Department of Dermatology, Seoul National University College of Medicine, 101, Daehak-ro, Jongno-gu, Seoul, Republic of Korea; 2 Division of Hematology/Oncology, Department of Pediatrics, Seoul National University College of Medicine, Seoul National University Children’s Hospital, 101, Daehak-ro, Jongno-gu, Seoul, Republic of Korea; 3 Seoul National University Cancer Research Institute, 101, Daehak-ro, Jongno-gu, Seoul, Republic of Korea; 4 Wide River Institute of Immunology, 101, Dabyeonbat-gil, Hwachon-myeon, Hongcheon-gun, Gangwon-do Republic of Korea; 5 Laboratory of Cutaneous Aging and Hair Research, Clinical Research Institute, Seoul National University Hospital, 101, Daehak-ro, Jongno-gu, Seoul, Republic of Korea; 6 Institute of Human-Environment Interface Biology, Seoul National University, 101, Daehak-ro, Jongno-gu, Seoul, Republic of Korea

**Keywords:** Skin diseases, Skin manifestations

## Abstract

Chemotherapy-induced alopecia (CIA) is a common and debilitating condition in children, with limited research on its characteristics and treatment. Therefore, this study aims to describe the characteristics of pediatric patients with CIA and the treatment outcomes of topical minoxidil and l-cystine, medicinal yeast, and pantothenic acid complex-based dietary supplements (CYP). This retrospective cohort study analyzed data from patients who underwent high-dose conditioning chemotherapy followed by hematopoietic stem cell transplantation and were treated with either topical minoxidil or CYP for CIA between January 2011 and January 2022. Among the 70 patients evaluated, 61 (87.1%) experienced clinical improvement. Patients in the groups with superior treatment outcomes received a greater cumulative amount of minoxidil and underwent treatment for a more extended duration (*P* < 0.05) than those in the other groups. All 70 (100%) patients received topical minoxidil, and 42 (60%) were administered CYP. Hair thickness was significantly higher in the combination therapy group than in the minoxidil monotherapy group (21.4% vs. 9.3%, *P* = 0.02). However, only 3 (4.3%) patients reported mild and self-limiting adverse events. In conclusion, our study shows that minoxidil and CYP administration represent viable treatment options for pediatric CIA.

## Introduction

Chemotherapy-induced alopecia (CIA) is a frequently encountered dermatological condition among individuals who have undergone cancer treatment, which may lead to psychological distress. Pediatric CIA is particularly distressing considering the social stigma attached to hair loss. Moreover, a subset of patients exhibits a chronic course with severe hair loss after 6 months post-chemotherapy, resulting in permanent CIA. Permanent CIA is usually noted after high-dose conditioning chemotherapy for hematopoietic stem cell transplantation (HSCT), treatment of high-risk or relapsed hematologic malignancies, and non-malignant hematologic diseases. Several studies indicate that over 90% of pediatric patients with cancer experience hair loss^1^, and the reported incidence of pediatric permanent CIA among pediatric CIA cases ranges between 12 and 24%^2,3^. Alopecia experienced by pediatric patients with cancer is not solely induced by chemotherapy but has a multifactorial nature influenced by factors, such as radiation and chronic cutaneous graft-versus-host disease.

Topical minoxidil, a United States Food and Drug Administration (FDA) approved medication, is a safe and effective treatment for regrowing hair in adults with patterned alopecia^4^. Several studies have reported its efficacy in adult CIA, even in cases of permanent CIA. Treatment with 5% topical minoxidil has shown more than moderate improvement in approximately 53–67% of patients^5,6^. l-cystine, medicinal yeast, and pantothenic acid complex-based dietary supplements (CYP) are commonly used over-the-counter products for hair loss treatment (Supplementary Table S1). Furthermore, several studies have revealed their benefits for treating diffuse telogen effluvium^7–10^.

Despite the high prevalence and negative impact of CIA, studies on the characteristics and treatment of pediatric CIA are limited. Therefore, this study aims to describe the clinical features of pediatric patients with CIA and evaluate the outcomes of combination therapy (topical minoxidil and oral CYP) and topical minoxidil monotherapy.

## Methods

### Study subjects and data collection

In this retrospective cohort study, we identified patients who visited the Pediatric Dermatology Department between January 2011 and January 2022 due to CIA. All patients had undergone high-dose conditioning chemotherapy followed by HSCT and were disease-free without therapy at their first visit to the Dermatology Department. We included patients treated with topical minoxidil or oral CYP for CIA, excluding those with recurrent underlying diseases or a diagnosis of other secondary diseases requiring chemotherapy or radiotherapy after initiating CIA treatment. We also excluded patients with incomplete medical records, insufficient clinical photographs, or other forms of alopecia, such as alopecia areata.

We collected demographic information and clinical data, including chemotherapy and underlying diseases, from the medical records, as well as details about CIA treatment and self-reported adverse reactions. The doses were measured in grams (g) and capsules for topical minoxidil and CYP, respectively. Topical minoxidil is available in 3% and 5% solution or foam, and 0.03 g and 0.05 g of minoxidil are calculated per application, respectively. A single capsule of CYP comprises 60, 20, 20, 100, 20, and 60 mg of calcium pantothenate, keratin, l-Cystine, medicinal yeast, p-Aminobenzoic acid, and thiamine nitrate, respectively. We prescribed 3 or 5% topical minoxidil and CYP once or twice daily after discussion with the patients and their parents. Additionally, the missed doses were identified using medical charts. The Institutional Review Board of Seoul National University Hospital approved the study protocol (IRB Number: 2104-059-1210), and its methods were performed in accordance with the principles of the Declaration of Helsinki. The requirement for individual patient consent was waived; we obtained informed consent for the open-access publication of clinical photographs from the parents of the markedly improved patients.

### Evaluation of hair regrowth

The efficacy of the treatment on CIA was evaluated using clinical photographs and phototrichogram (Folliscope 5.0, LeadM, Korea) data obtained at baseline and the last visits. The visit was considered the last visit in cases where the clinician planned for self-follow-up without scheduling further appointments or the patients did not return for an additional visit. In cases where patients arbitrarily discontinued topical or oral medications during treatment, the visit immediately preceding discontinuation was considered the last visit. Standardized scalp photographs obtained from fronto-mid and vertex views were reviewed by two independent dermatologists who used the Common Terminology Criteria for Adverse Events (CTCAE), version 5.0, to grade the extent of hair loss. For grading CIA, a hair loss of < 50% of the normal level that does not require a wig or hairpiece for camouflage is considered as grade 1^11^, while a hair loss of > 50% of the normal level that requires camouflage or is associated with psychosocial impact is categorized as grade 2. The treatment outcome for each patient was categorized into four groups based on a comparison of the baseline and final grades as follows^12^: complete response (CR), partial response (PR), stable disease (SD), and progression of disease (PD) indicate improvement of alopecia with a decrease in CTCAE grade, improved alopecia without a reduction of CTCAE grade, no change in scalp coverage, and worsening alopecia, respectively. In accordance with the criteria established in a previous study^3^, extensive or complete hair loss (> 75%) lasting for at least 6 months post-chemotherapy discontinuation was considered permanent pediatric CIA.

Two adjacent scalp sites on the vertex, located at the crosspoint of the sagittal line and the line connecting the external auditory meatus, were used to obtain phototrichograms. The hair density and thickness were measured using image analysis software (ImageJ, Version 1.24; National Institutes of Health, Baltimore, MD, USA) under 50- and 100-fold magnification, respectively. The total number of hair shafts in a 1 cm^2^ circular area was manually counted to determine the hair density. Additionally, the thickness of the thickest five hairs in a 1 cm^2^ area was measured, and their average was calculated to obtain the hair thickness. Finally, the measurements obtained at each site were averaged.

### Statistical analysis

Patient characteristics were summarized and analyzed descriptively. CIA treatment variables, including the medication type and its cumulative dose and the duration and latency of treatment, were compared among the four treatment outcome groups. Additionally, chemotherapy variables, such as the underlying disease and regimen, were also compared among the four groups. Hair regrowth, as indicated by an increase in hair density and thickness, was compared between the groups administered topical minoxidil alone and topical minoxidil plus CYP.

Continuous variables were analyzed using a one-way analysis of variance or the Kruskal–Wallis H test and were reported as mean and standard deviations (SDs) or median and interquartile ranges (IQRs). Categorical variables were compared using the chi-square or Fisher’s exact test. All statistical analyses were performed using IBM SPSS Statistics for Windows, version 25.0 (IBM Corp., Armonk, N.Y., USA). We used Benjamini–Hochberg correction to control the false discovery rate in multiple comparisons. A *P* value < 0.05 in two-sided tests was considered statistically significant.

### Ethics statement

Reviewed and approved by Seoul National University Hospital (IRB number: 2104-059-1210).

### Patient consent

Consent for the publication of recognizable patient photographs or other identifiable material was obtained by the authors and included at the time of article submission to the journal, stating that all patients gave consent with the understanding that this information may be publicly available.

## Results

### Characteristics of patients with chemotherapy-induced alopecia

Overall, 70 patients with a mean age at HSCT of 8.6 ± 5.7 years were included in this study (Table 1), and 31 (44.3%) were males. The most common underlying disease requiring HSCT was leukemia (n = 32, 45.7%). Among the 26 chemotherapy regimens used, the most frequently employed was the combination of busulfan, fludarabine, etoposide, and thymoglobulin (BuFluVPATG), used in 8 (11.4%) patients. Of the 10 patients who underwent cranial irradiation before the initiation of hair loss treatment, cumulative doses of cranial irradiation were identified in 7 patients by reviewing medical charts. The average cumulative dose for these patients was 48.3 Gy (ranging from 48.6 to 54 Gy), which is higher than the permanent radiation-induced alopecia threshold reported in pediatric patients^13^. Nine (12.9%) patients had permanent CIA. The median latency between HSCT and CIA treatment initiation was 445 (IQR: 316.8–698.5) days, approximately 64 weeks. Furthermore, the patients maintained their treatment for a median duration of 317 (IQR: 178.8–578) days, which is approximately 45 weeks. The median cumulative dose of CYP supplement and topical minoxidil was 167.5 (IQR: 0–551) capsules and 12.5 (IQR: 6.6–27.1) g, respectively.

**Table 1:** Characteristics of patients with pediatric chemotherapy-induced alopecia (n = 70).

*Demographics and underlying disease*	
Age at HSCT, mean ± SD, y	8.6 ± 5.7
Male patients, n (%)	31 (44.3)
Diagnosis, n (%)	
Leukemia	32 (45.7)
Solid tumor	21 (30)
Brain tumor	10 (14.3)
Non-malignant disease*	5 (7.1)
Lymphoma	2 (2.9)
Type of HSCT, n (%)	
Allograft	40 (57.1)
Autograft	30 (42.9)
History of cranial irradiation, n (%)	10 (14.3)
History of graft-versus-host disease, n (%)	17 (24.3)
*Chemotherapy-induced alopecia*	
Permanent CIA, n (%)	9 (12.9)
Latency of treatment, median (IQR), d	445 (316.8–698.5)
Duration of treatment, median (IQR), d	317 (178.8–578)
Cumulative dose of CYP supplement, median (IQR), capsule	167.5 (0–551)
Cumulative dose of topical minoxidil, median (IQR), g	12.5 (6.6–27.1)
Severity grade of alopecia^†^	
Grade 1 at baseline, n (%)	35 (50)
Grade 1 at last follow-up, n (%)	46 (65.7)
Hair density	
Baseline, mean ± SD/cm^2^	132.5 ± 39.9
Last follow-up, mean ± SD/cm^2^	166 ± 41.6
Percentage increase from baseline, %	30.2
Hair thickness	
Baseline, mean ± SD, nm	49.6 ± 9.8
Last follow-up, mean ± SD, nm	51.9 ± 19.7
Percentage increase from baseline, %	14.6

*SD*, standard deviation; *y*, years; *HSCT*, hematopoietic stem cell transplantation; *n*, number; *CIA*, chemotherapy-induced alopecia; *CYP*, l-cystine, medicinal yeast, and pantothenic acid complex-based dietary supplement; *IQR*, interquartile range; d, days.

*Chronic granulomatous disease (3), severe aplastic anemia (1), and Wiskott–Aldrich Syndrome (1) were included.

^†^Graded by Common Terminology Criteria for Adverse Events, version 5.0. Grade 1 is hair loss < 50% of normal, which does not require a wig or hair piece to camouflage. Grade 2 is hair loss > 50% of normal requiring camouflage or associated with psychosocial impact.

**Figure 1:**
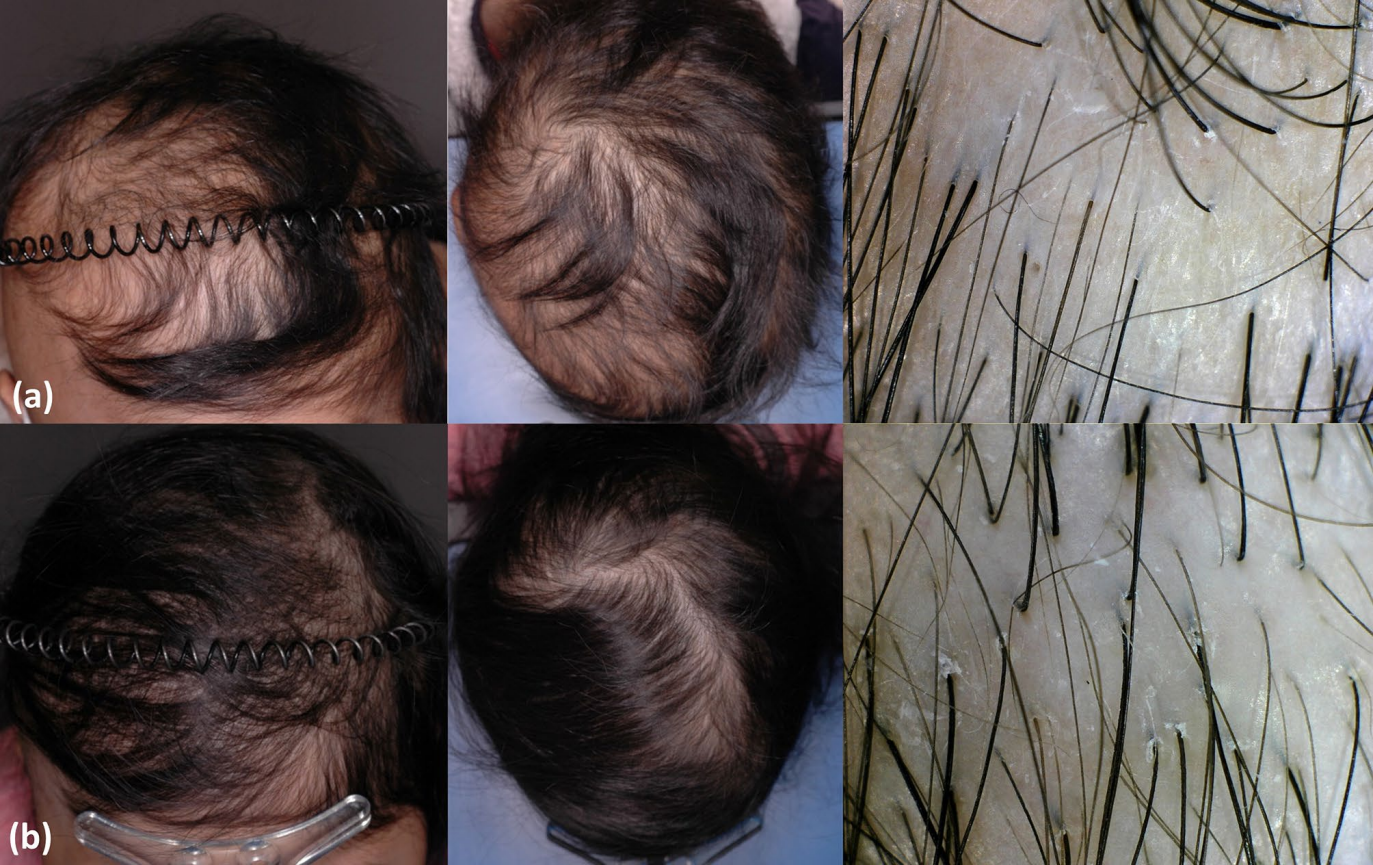
“Improved pediatric chemotherapy-induced alopecia after topical minoxidil application and dietary supplementation.” An 8-year-old female patient was diagnosed with primitive neuroectodermal tumors and administered high-dose chemotherapy followed by hematopoietic stem cell transplantation (HSCT). (**a**) Clinical photographs (left, fronto-mid view; middle, vertex view) and trichoscope view (right, 50 ×) of the first visit (10 months after HSCT). (**b**) Clinical photographs (left, fronto-mid view; middle, vertex view) and trichoscope view (right, 50 ×) after treatment for 20 months.

Most patients experienced obvious clinical improvement except for nine (12.9%) patients with stable disease. Complete and partial responses were achieved in 11 (15.7%) and 50 (71.4%) patients, respectively. Figure 1 shows clinical photographs and phototrichogram images of a patient with a complete response. Additionally, hair density and thickness, which are objective parameters, increased by a mean of 30.2% and 14.6%, respectively.

Only three patients reported adverse events: mild burning sensation, itching, and erythema after application of 3% topical minoxidil, and a prickling sensation after administration of CYP. Although these symptoms resolved spontaneously in a week, the medication was discontinued based on parental preferences.

### Comparison of clinical characteristics and treatment of chemotherapy-induced alopecia by treatment outcome

Regarding the comparison of treatment details among the three groups with different treatment outcomes (Table 2), the median cumulative dose of topical minoxidil was higher in the groups with better treatment outcomes than in those without (26.2, 14, and 4.9 g in the complete response, partial response, and stable disease groups, respectively; adjusted *P* = 0.008). Although the median cumulative dose of CYP was also observed to be higher in the groups with superior responses than in those without, this trend did not reach statistical significance. Patients who demonstrated better treatment outcomes continued treatment for longer durations than those who did not (median: 626, 317, and 154 days in the complete response, partial response, and stable disease groups, respectively; adjusted *P* = 0.001). No statistically significant differences were noted in demographics, such as age and sex, or underlying disease characteristics, including diagnosis, treatment, and history of graft-versus-host disease.

**Table 2:** Clinical characteristics and treatment of pediatric chemotherapy-induced alopecia by treatment outcome.

	*Complete response (n = 11)*	*Partial response (n = 50)*	*Stable disease (n = 9)*	*P* value*
*Demographics and underlying disease*				
Age at HSCT, mean ± SD, y	8.1 ± 6	8.8 ± 6	8 ± 4.4	1
Male patients, n (%)	6 (54.5)	20 (40)	5 (55.6)	0.825
Underlying disease, n (%)				0.69
Leukemia	3 (27.3)	25 (50)	4 (44.4)	
Solid tumor	4 (36.4)	15 (30)	2 (22.2)	
Brain tumor	1 (9.1)	7 (14)	2 (22.2)	
Non-malignant disease	2 (18.2)	2 (4)	1 (11.1)	
Lymphoma	1 (9.1)	1 (2)	0	
Type of HSCT, n (%)				1
Allograft	7 (63.6)	29 (58)	5 (55.6)	
Autograft	4 (36.4)	21 (42)	4 (44.4)	
History of myeloablative agent, n (%)				
Thiotepa	1 (9.1)	8 (16)	2 (22.2)	0.95
Busulfan	9 (81.8)	35 (70)	4 (44.4)	0.9
Cyclophosphamide	2 (18.2)	5 (10)	2 (22.2)	0.814
History of cranial irradiation, n (%)	2 (18.2)	6 (12)	2 (22.2)	0.873
History of graft-versus-host disease, n (%)	0	2 (4)	1 (11.1)	0.769
*Chemotherapy-induced alopecia*				
Treatment				.93
Topical minoxidil only	2 (18.2)	22 (44)	4 (44.4)	
Topical minoxidil + CYP supplement	9 (81.8)	28 (56)	5 (55.6)	
Cumulative dose of topical minoxidil, median (IQR), g	26.2 (12.3–59.4)	14 (6.9–28)	4.9 (2.4–7.8)	0.008^†^
Cumulative dose of CYP supplement, median (IQR), cap	672 (243–1973)	152.5 (0–435.5)	98 (0–307)	0.08
Latency of treatment, median (IQR), d	396 (300–501)	445 (302.5–716.5)	473 (308–1431.8)	.933
Duration of treatment, median (IQR), d	626 (438–1215)	317 (182.5–554)	154 (58.5–220)	.001^†^

*SD*, standard deviation; *y*, years; *HSCT*, hematopoietic stem cell transplantation; *n*, number; *CYP*, ;l-cystine, medicinal yeast, and pantothenic acid complex-based dietary supplement; *IQR*, interquartile range; *cap*, capsule; d, days.

*Corrected for multiple comparisons with the Benjamini–Hochberg procedure.

^†^Denotes statistical significance.

### Improved hair parameters and comparison by type of treatment

**Figure 2:**
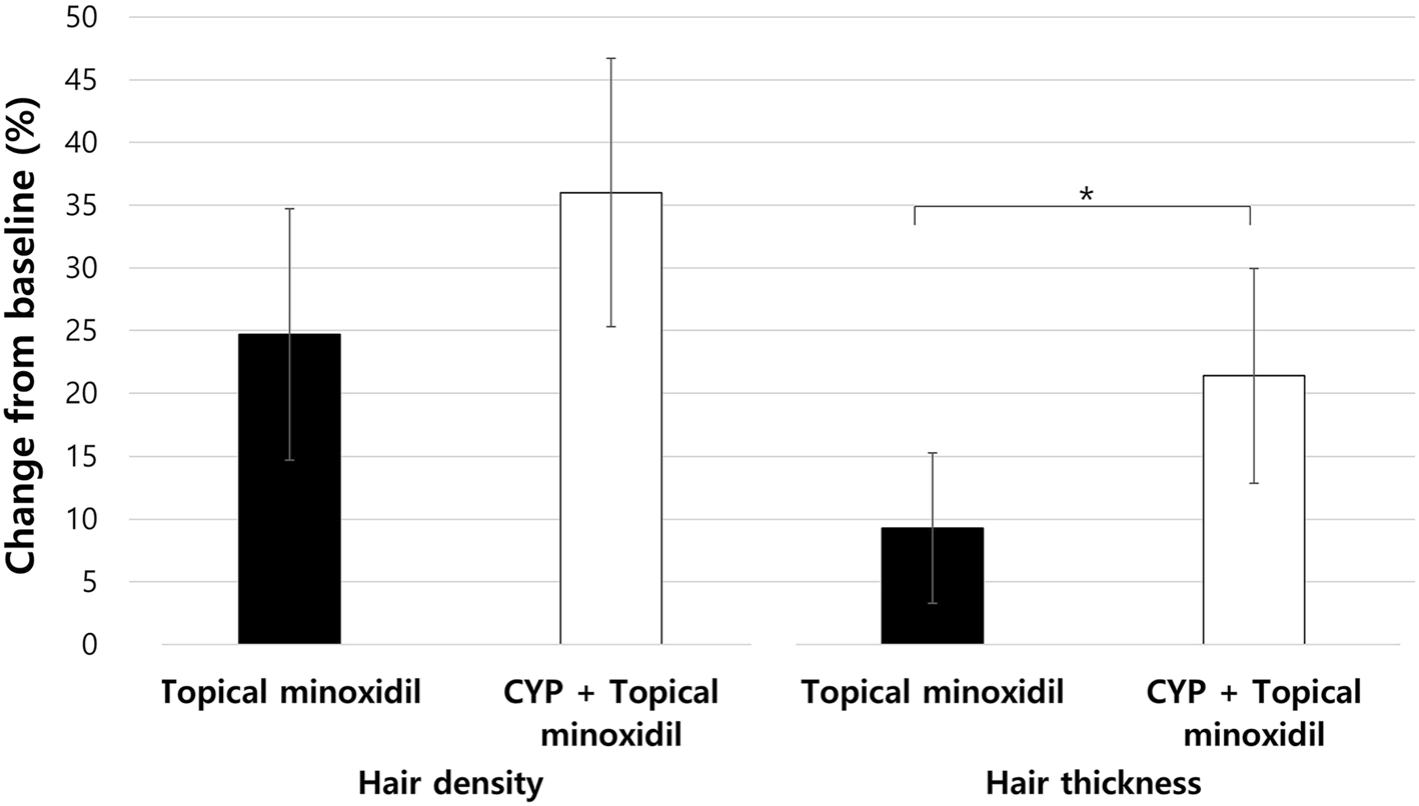
“Comparison of % change of hair growth parameters (density or thickness) in pediatric chemotherapy-induced alopecia between monotherapy and combination therapy group.” Better treatment outcome was noted in the combination therapy group (CYP + topical minoxidil) than in the topical minoxidil monotherapy group (for density, 36% vs. 24.7%, *P* = 0.153; for thickness, 21.4% vs. 9.3%, *P* = 0.02). Error bars represent 95% confidence intervals. *CYP*, l-cystine, medicinal yeast, and pantothenic acid complex-based dietary supplement.

All patients included in this study were treated with topical minoxidil, and 42 (60%) of them also received CYP. No significant difference was found in the baseline characteristics of minoxidil monotherapy and combination therapy groups (Table 3). Our findings, as shown in Fig. 2, indicate that both groups exhibited improvements in hair density and thickness. However, patients who received combination therapy had significantly greater hair thickness than those who received topical minoxidil monotherapy (21.4% vs. 9.3%, *P* = 0.02). Hair density tended to be higher in patients who received combination therapy than in those who received topical minoxidil monotherapy, without significant difference (36% vs. 24.7%, *P* = 0.153).

**Table 3:** Baseline characteristics of minoxidil monotherapy and combination therapy groups.

	*Topical minoxidil (n = 28)*	*CYP + Topical minoxidil (n = 42)*	*P* value*
*Demographics and underlying disease*			
Age at HSCT, mean ± SD, y	9 ± 6.3	7.8 ± 5.3	1
Male patients, n (%)	12 (42.9)	19 (45.2)	1
Underlying disease, n (%)			1
Leukemia	15 (53.6)	17 (40.5)	
Solid tumor	7 (25)	14 (33.3)	
Brain tumor	3 (10.7)	7 (16.7)	
Non-malignant disease	2 (7.1)	3 (7.1)	
Lymphoma	1 (3.6)	1 (2.4)	
Type of HSCT, n (%)			0.56
Allograft	20 (71.4)	21 (50)	
Autograft	8 (28.6)	21 (50)	
History of myeloablative agent, n (%)			
Thiotepa	2 (7.1)	9 (21.4)	.5
Busulfan	21 (75)	27 (64.3)	0.76
Cyclophosphamide	4 (14.3)	5 (11.9)	1
History of cranial irradiation, n (%)	2 (7.1)	1 (2.4)	0.69
History of graft-versus-host disease, n (%)	10 (35.7)	7 (16.7)	0.32
*Chemotherapy-induced alopecia*			
Permanent CIA, n (%)	3 (10.7)	6 (14.3)	1
Latency of treatment, median (IQR), d	611.6 ± 488.4	562 ± 436.4	1
Severity grade of alopecia^†^			
Grade 1 at baseline, n (%)	18 (64.3)	17 (40.5)	0.41
Baseline hair density, mean ± SD/cm^2^	136 ± 39.3	126.9 ± 36	0.74
Baseline hair thickness, mean ± SD, nm	52.5 ± 9.8	47.5 ± 9.6	1

*SD*, standard deviation; *y*, years; *HSCT*, hematopoietic stem cell transplantation; *n*, number; *CYP*, ;l-cystine, medicinal yeast, and pantothenic acid complex-based dietary supplement; *CIA*, chemotherapy-induced alopecia; *IQR*, interquartile range; *cap*, capsule; d, days.

*Corrected for multiple comparisons with the Benjamini–Hochberg procedure.

^†^Graded by Common Terminology Criteria for Adverse Events, version 5.0. Grade 1 is hair loss < 50% of normal, which does not require a wig or hair piece to camouflage. Grade 2 is hair loss > 50% of normal requiring camouflage or associated with psychosocial impact.

## Discussion

This study examined the clinical features of 70 pediatric patients with CIA and evaluated the effectiveness of topical minoxidil and dietary supplements as treatment options. Our results revealed that approximately 87% of the patients exhibited enhanced scalp coverage, as evidenced by increased hair density and thickness. Additionally, the permanent CIA rate in our study was 12.9%, which is consistent with the findings of a previous study^3^ that used identical diagnostic criteria (12%).

Chemotherapeutic agents can directly harm hair matrix cells, resulting in CIA. CIA is known as a type of anagen effluvium since hairs in the highly proliferative anagen phase are affected^14^. Minoxidil is known to modulate the hair cycle by inducing and prolonging the anagen stage^15^, which is attributed to its sulfated metabolite’s action as a potassium channel opener^16^. Topical or oral minoxidil has been proven effective and safe in previous reports for treating adult CIA^5,6,17–21^. Our study also revealed the dose–response relationship between topical minoxidil and treatment outcomes in pediatric CIA. Similar to the senescence process, where depletion and decreased activity of hair follicle stem cells prolongs the telogen period^22^, chemotherapy can also damage hair follicle stem cells and delay the transition from telogen to anagen. Therefore, CIA has a component of telogen effluvium rather than a pure anagen effluvium^23^. We suggest that CYP, reported with improved telogen effluvium, may also confer benefits in the treatment of CIA, which has a complex nature. Recent in vitro studies have shown that the core compounds of CYP, particularly l-cystine, positively impact cell proliferation, viability, and the protection of human hair follicular keratinocytes (HHFKs) and support fundamental cellular processes relevant to healthy hair growth. By quantifying hair parameters, we identified the beneficial additive effects of CYP in treating pediatric CIA.

In our study, we found that better treatment outcomes for pediatric CIA were associated with a longer duration of treatment, higher cumulative dose of medication, and combination therapy. These factors are closely linked to adherence to a prescription with an active attitude approach towards well-being. Additionally, patients and caregivers may have engaged in lifestyle modifications beneficial for hair loss treatment, such as avoiding hair traction and carefully washing and drying hair. The importance of adhering to treatment in therapeutic outcomes has been emphasized not only in chronic systemic disorders, such as hypertension and diabetes^24,25^, but also in chronic dermatological disorders, including psoriasis and atopic dermatitis^26^. Therefore, to address poor adherence to topical treatment compared to oral medication^27–29^, low-dose oral minoxidil has recently emerged as a preferred option for various patterns of hair loss.

Special consideration should be given to the potential systemic adverse effects of CIA treatment since pediatric patients with CIA have a history of malignancy. Despite not being FDA-approved for patients aged < 18 years, minoxidil has been used off-label for hair loss in children with a tolerable safety profile, in topical or oral form^30–33^. The most common adverse events of topical minoxidil include contact dermatitis, transient shedding, and localized or generalized hypertrichosis^34^. Although three reports of transient palpitations and dizziness are available, systemic absorption has been suggested to be minimal when complying with the prescribed usage, with < 2% of the applied dose being absorbed^35^. While no previous study has investigated the use of CYP for treating pediatric patients, CYP compounds, which are naturally occurring substances found in plants and animals, are considered safe at commercial doses. Some previous studies conducted with adults have reported mild gastrointestinal discomfort and weight gain^7,10^. In this study, three patients experienced mild cutaneous adverse events that resolved spontaneously. Furthermore, two of these patients were treated with topical minoxidil, while one received CYP, and none experienced systemic adverse events.

This study has some limitations. First, the absence of a control group without treatment limited our ability to distinguish between the treatment effect and natural recovery. Most pediatric patients with CIA typically experience full hair regrowth within 6 months^36^. However, in this study, all patients, except three, started alopecia treatment 6 months after the end of chemotherapy with incomplete hair regrowth. Second, the patients were followed up for a median of 317 days, which did not allow for the investigation of the long-term safety of treatment and maintenance of treatment outcomes. Particularly, CYP, whose safety in pediatric populations has not been addressed in previous studies, should be administered after consultation with physicians. Therefore, further studies with a randomized controlled design and longer follow-up duration are necessary to clarify the efficacy, persistence, and long-term safety of minoxidil and CYP in pediatric patients with CIA.

In conclusion, this retrospective study showed that topical minoxidil was associated with improved clinical outcomes in pediatric patients with CIA. Additionally, dietary supplementation with CYP might have provided further benefits for these patients. Both topical minoxidil and CYP were well-tolerated with rare and mild adverse events. While acknowledging the need for further prospective studies, our findings suggest that minoxidil and CYP could be feasible treatment options for pediatric CIA. Furthermore, adherence to treatment is a crucial factor in achieving successful outcomes in the CIA treatment.

### Supplementary Information

Supplementary Information.

## Data Availability

Supporting data on the findings of this study are available on request from the corresponding author. The data are not publicly available due to privacy and ethical restrictions.
