# Supplementary material for: Topical minoxidil and dietary supplement for the treatment of chemotherapy-induced alopecia in childhood: a retrospective cohort study
Source: Sci Rep. 2024 Feb 22;14:4349. doi: 10.1038/s41598-024-53054-8 (PMC10884397; doi:10.1038/s41598-024-53054-8)
Supplement: Supplementary file 1 — Supplementary Information. [file 41598_2024_53054_MOESM1_ESM.docx]

Supplementary table 1. List of L-cystine, medicinal yeast, and pantothenic acid complex-based dietary supplements (CYP) products*

| ***Product Name*** | ***Manufacturer (Country)*** |
| --- | --- |
| Pantogar® | Merz Therapeutics (Germany) |
| Pantovigar® | Merz Therapeutics (Germany) |
| Minoxyl S® | Hyundai Pharm (Republic of Korea) |
| Pansidil® | Dongkook Pharmaceutical (Republic of Korea) |
| Capillus capsule® | Dong-A Pharmaceutical (Republic of Korea) |

* The products are listed when their ingredient quantities are known and same with Pantogar®
